# Tissue miR-200c-3p and circulating miR-1290 as potential prognostic biomarkers for colorectal cancer

**DOI:** 10.1038/s41598-022-06192-w

**Published:** 2022-02-10

**Authors:** Enoch Kang, Sung Cheol Jung, Soo Kyung Nam, Yujun Park, Soo Hyun Seo, Kyoung Un Park, Heung-Kwon Oh, Duck-Woo Kim, Sung-Bum Kang, Hye Seung Lee

**Affiliations:** 1 Seoul National University College of Medicine, 103 Daehak-ro, Jongno-gu, Seoul, 03080 Republic of Korea; 2 Department of Pathology, Seoul National University College of Medicine, Seoul, 03080 Republic of Korea; 3 Department of Pathology, Seoul National University Bundang Hospital, 173-82 Gumi-ro, Bundang-gu, Seongnam-si, Gyeonggi-do 13620 Republic of Korea; 4 Department of Laboratory Medicine, Seoul National University Bundang Hospital, Seongnam-si, Gyeonggi-do 13620 Republic of Korea; 5 Department of Laboratory Medicine, Seoul National University College of Medicine, Seoul, 03080 Republic of Korea; 6 Department of Surgery, Seoul National University Bundang Hospital, Seongnam-si, Gyeonggi-do 13620 Republic of Korea; 7 Department of Surgery, Seoul National University College of Medicine, Seoul, 03080 Republic of Korea; 8 Department of Pathology, Seoul National University Hospital, 101 Daehak-ro, Jongno-gu, Seoul, 03080 Republic of Korea

**Keywords:** Gastrointestinal cancer, Prognostic markers, Non-coding RNAs

## Abstract

Epithelial–mesenchymal transition (EMT)-related cancers generally elicit low immune responses. EMT is regulated by several microRNAs (miRNAs) in cancers. Thus, this study aimed to evaluate the prognostic potential of EMT-related miRNAs as biomarkers in colorectal cancer (CRC). Formalin-fixed paraffin-embedded tumor and normal tissue and plasma samples were obtained from 65 patients with pathologically confirmed CRC. In addition, plasma samples were obtained from 30 healthy volunteers. Immunohistochemical staining for E-cadherin, ZEB1, PD-1, PD-L1, CD3, CD4, CD8, Foxp3, and CD68 was conducted on tissue samples. Droplet digital polymerase chain reaction (ddPCR) analysis was performed to evaluate miR-21-5p, 34a-5p, 138-5p, 200a-3p, 200b-5p, 200c-3p, 630, 1246, and 1290 expression in tissue samples and miR-630, 1246, and 1290 expression in plasma samples. miR-21-5p, 34a-5p, 630, 1246, and 1290 expression was higher in tumor tissues than in normal tissues (*P* < 0.05). EMT was significantly associated with reduced tumor-infiltrating T cells. Moreover, miR-21-5p, miR-34a-5p, miR-200a-3p, and miR-200c-3p expression was negatively correlated with T cell density (*P* < 0.05). High tissue levels of miR-200c-3p were associated with poor overall survival (OS) (*P* < 0.001). CRC patients with the EMT phenotype had poor OS; however, PD-L1 positivity and abundant PD-1 positive immune cells were correlated with better OS (P < 0.05). miR-1246 and miR-1290 levels were significantly higher in the plasma of patients with CRC than in the plasma of healthy controls (*P* < 0.05). High plasma levels of miR-1290 were correlated with advanced stage and poor OS (*P* < 0.05). The tissue expression of miR-200c-3p and plasma levels of miR-1290 measured by ddPCR indicate their potential as prognostic biomarkers for CRC.

## Introduction

Colorectal cancer (CRC) contributes significantly to the global cancer burden, ranking third in incidence and second in mortality^1^. In recent years, advances in diagnostic and therapeutic strategies have resulted in a decrease in the incidence of CRC and improvement in patient survival^2^. However, the treatment of metastatic CRC remains a considerable challenge^3^. With the development of new chemotherapeutic and immunotherapeutic agents, the cost of CRC treatment has increased considerably, while survival rates have remained limited. Multiagent approaches have been developed based on availability and not on the basis of validated and refined treatment algorithms^4^. To overcome such challenges, it is necessary to identify biomarkers that enable accurate prognosis and a personalized approach in the treatment of CRC.

The epithelial–mesenchymal transition (EMT) is a key cellular process in CRC progression and metastasis^5^. Molecular pathways (including EMT) vary widely, and different investigators have used different methods to classify EMT. Several commonly used EMT markers include the loss of E-cadherin expression and increased expression of EMT-related transcription factors, such as ZEB1^6,7^. Recent findings have suggested that relationships may exist between EMT and microRNAs (miRNAs)^8^ or tumor-associated immune cells^9^.

miRNAs are non-coding RNAs comprising 20 to 22 nucleotides that regulate gene expression^10^. Several studies suggest that miRNAs are involved in cancer progression, because miRNAs regulate the expression of tumor-suppressor genes, oncogenes, and other regulatory molecules involved in cell differentiation, apoptosis, and tumorigenesis^11–13^. Furthermore, recent reports show that miRNAs are involved in EMT regulation. miR-21-5p, an important miRNA in cancer, is located on chromosome 17q23.2, which frequently has a copy-number gain in metastatic CRCs^14^. Downregulation of miR-21-5p has been reported to reverse EMT and the cancer stem cell phenotype^15^. Moreover, the miR-200 family has been reported to target and downregulate ZEB1, an EMT activator^16^. However, these EMT-related miRNAs likely have several different targets and function at various levels.

The tumor immune microenvironment is another key factor in CRC progression and metastasis^17^. With an increasing interest in immunotherapy, targeting the tumor immune microenvironment has emerged as a therapeutic strategy. Previous findings have suggested that the immunological synapse between PD-1 (which is expressed on lymphocytes) and PD-L1 (which is expressed on tumor cells) causes cytotoxic T cell anergy in the tumor microenvironment, enabling further tumor progression^11^. miRNAs are also known to contribute to immune evasion of neoplastic cells through the regulation of various pathways^18^, as well as PD-1 and PD-L1 expression^19^. Thus, miRNAs have the potential to serve as diagnostic and prognostic markers.

Although miRNAs are typically expressed in tissue samples, they can also be detected in blood samples as they are released from tumor cells into the circulation^20^. Despite their small quantity, circulating miRNAs have apparent merits over tissue miRNAs as biomarkers because blood samples are easier and less invasive to obtain than tissue samples. Therefore, analysis of circulating miRNAs in addition to that of tissue miRNAs is necessary to assess their potential as novel biomarkers.

Although the roles of the EMT, tumor immune microenvironment, and related miRNAs in CRC progression have been widely studied, the clinical significance of the related miRNAs remains unclear. This is partly owing to the extensive number of targets and functional roles of miRNAs. In addition, studies involving human subjects are rare. Consequently, this study aims to illustrate the relationship among miRNAs, EMT, and the tumor immune microenvironment in CRC and determine the clinical potentials of several miRNAs as prognostic biomarkers. We quantified the expression levels of miRNAs previously reported to be related to EMT in various cancers, using tissue and plasma samples from 65 patients with CRC. We then investigated the clinicopathologic significance of the measured miRNA expression levels.

## Methods

### Study population and clinical specimens

This study included plasma specimens from 30 healthy blood donors and tissue and plasma specimens from 65 patients with CRC who underwent radical surgical resection at the Seoul National University Bundang Hospital between March 2011 and March 2012. Patients who received preoperative radiotherapy or chemotherapy were excluded from the study. Clinicopathologic features of CRC patients are summarized in Table 1. Tissue specimens were obtained during resection, whereas plasma samples were obtained approximately 1 to 20 days before resection. Tissue samples were fixed in 4% buffered formalin solution and embedded in paraffin. Blood samples were processed within 2 h of collection and centrifuged at 3,000 rpm for 10 min. Each plasma sample was filtered through a Fisherbrand Standard Serum Filter (13 mm × 4″; Fisher HealthCare, Houston, TX, USA) and stored at -80 °C until use. Clinicopathologic data such as age, sex, histological grade, and patients’ overall survival (OS) were obtained from electronic medical records. OS was defined as the period from surgery to death from any cause or to the date of the last follow-up. Cancer stages were determined based on the guidelines from the American Joint Committee on Cancer (8th edition).

**Table 1:** Summary of clinicopathologic features of patients with colorectal cancer.

Variable	N (%)
**Mean age (Range)-year**	64.7 (38–85)
**Sex**	
Male	40 (61.5)
Female	25 (38.5)
**Tumor size**	
< 5 cm	44 (67.7)
≥ 5 cm	21 (32.3)
**Histologic grade**	
WD	8 (12.3)
MD	49 (75.4)
PD	8 (12.3)
**Lymphatic invasion**	
Absent	43 (66.2)
Present	22 (33.8)
**Venous invasion**	
Absent	49 (75.4)
Present	16 (24.6)
**Perineural invasion**	
Absent	39 (60)
Present	26 (40)
**TNM staging**	
I	17 (26.2)
II	16 (24.6)
III	16 (24.6)
IV	16 (24.6)
**Location**	
Right	14 (21.5)
Left/rectum	51 (78.5)
**KRAS**	
Wild	32 (49.2)
Mutant	33 (50.8)

*WD* well differentiated, *MD* moderately differentiated, *PD* poorly differentiated.

### Ethics approval and consent to participate

This study was approved by the Institutional Review Board of Seoul National University Bundang Hospital (approval number: B-1012/117–011) and performed in accordance with the tenets of the Declaration of Helsinki. Informed consent was obtained from all patients and healthy volunteers for the use of their clinical information and samples for analysis.

### Quantification of miRNAs by droplet digital polymerase chain reaction (ddPCR) analysis

Total RNA was extracted from paired normal and tumor formalin-fixed paraffin-embedded (FFPE) tissue samples. Four 8-µm-thick FFPE tissue sections were used for RNA extraction. Tissue sections were deparaffinized by incubation at 70 °C for 10 min and centrifugation for 10 min at maximum speed. After deparaffinization, RNA extraction was performed using RecoverAll™ Total Nucleic acid Isolation Kit (Invitrogen, Waltham, MA, USA) according to the manufacturer’s instructions. For blood samples, total RNA was extracted using a High Pure Viral Nucleic Acid Kit (Roche, Indianapolis, IN, USA) according to the manufacturer’s instructions with 300 µL of plasma.

Reverse transcription (RT) reactions were performed using the TaqMan™ MicroRNA Reverse Transcription Kit (200 reactions, catalog #4,366,596; Applied Biosystems, Waltham, MA, USA) for nine EMT-related miRNAs including miR-21-5p (Assay ID 000,397), miR-34a-5p (Assay ID 000,426), miR-138-5p (Assay ID 002,284), miR-200a-3p (Assay ID 000,502), miR-200b-5p (Assay ID 002,274), miR-200c-3p (Assay ID 001,563), miR-630 (Assay ID 001,563), miR-1246 (Assay ID CTPRJ9P), and miR-1290 (Assay ID 002,863). These miRNAs have been reported to regulate EMT or to function in relation to EMT^14,21–24^. Briefly, each reverse transcriptase reaction contained 7 µL of the RT mixture, 5 µL of total RNA (1 ng of total tissue RNA or 5 µL of plasma RNA), and 3 µL of 5 × RT primer. Each 15-µL reaction mixture was incubated in a C1000Touch™ Thermal Cycler (Bio-Rad, Hercules, CA, USA) at 16 °C for 30 min, 42 °C for 30 min, and 85 °C for 5 min.

miRNA expression levels were measured via ddPCR. Each ddPCR mixture contained 10 µL of ddPCR Supermix for Probes, 1 µL template DNA synthesized from RT reactions, 1 µL of a FAM-labeled probe for the miRNA of interest, and 8 µL of distilled water. Oil drops were generated using Droplet Generation Oil for Probes (Bio-Rad; catalog #1,863,005). The C1000 Touch™ Thermal Cycler, equipped with a deep-well block, was used for PCR analysis with the following thermocycling conditions: 95 °C for 10 min, 40 cycles of 94 °C for 30 s and 60 °C for 1 min, and 98 °C for 10 min. All data were interpreted using the Bio-Rad QX200 droplet reader and analyzed using the QuantaSoft program (version 1.7.4). The representative results of ddPCR fluorescence plots were shown in Additional file 1. Quantification was performed by determining the copy number of target miRNA/1 ng total RNA for tissue samples or the number of copies of target miRNA/1 µL cDNA for plasma samples, as previously described^25,26^.

### Tissue microarray (TMA) construction and immunohistochemistry (IHC)

A representative tissue core with a 2-mm diameter was obtained from each patient as an FFPE block, and sets of TMA blocks were made of these tissue cores. TMA blocks were constructed from the tumor center (TC) and the invasive margin (IM) of the tumor tissue. Using an automatic immunostainer (BenchMark XT; Ventana Medical Systems, Tucson, AZ, USA), the tissue samples were stained with antibodies for E-cadherin (mouse monoclonal; BD Biosciences, San Jose, CA, USA), ZEB1 (rabbit polyclonal; Novus Biologicals, Centennial CO, USA), CD3 (rabbit polyclonal; Dako, Carpinteria, CA, USA), CD4 (rabbit monoclonal; Ventana Medical Systems), CD8 (rabbit monoclonal; Ventana Medical Systems), CD68 (mouse monoclonal; Dako), Foxp3 (mouse monoclonal; Abcam, Cambridge, UK), PD-1 (rabbit monoclonal; Cell Signaling Technology, Danvers, MA, USA), and PD-L1 (mouse monoclonal; 22C3 PharmDx Kit; Dako). The stained images were digitalized for analysis.

For E-cadherin IHC, membranous expression was considered to reflect a positive result, and the area (%) of positive staining was examined. The area (%) and intensity of nuclear ZEB1 expression were recorded, and the intensities were classified as indicating negative (0), weak (1), or strong (2) expression. EMT phenotype was defined as any loss of E-cadherin expression or a score greater than 20 as determined by multiplying the ZEB1-expression intensity by the % area positive for ZEB1 expression.

PD-L1 was interpreted with a combined positive score (CPS). CPS is defined as the percentage of PD-L1 staining cells (tumor cells, lymphocytes, and macrophages) relative to total viable tumor cells. CPS ≥ 1 was used as the cut-off for PD-L1 positive^27^.

### Image analysis for immune cell densities

The digitalized IHC-stained images were analyzed using QuPath software (version 0.2.0-m4). For each IHC-stained image for the immune checkpoint marker, PD-1, or immune cell markers (CD3, CD4, CD8, CD68, and Foxp3), the number of positively stained cells was counted using the cell count function in QuPath, and the density was calculated as the number of cells/mm^2^.

### KRAS mutation analysis using the PNAClamp™ KRAS Mutation Detection Kit

DNA was extracted from tumor tissues obtained from 65 patients with CRC, using the QIAamp® DNA FFPE Tissue Kit (Qiagen, Hilden, Germany). KRAS mutations in the CRC tissue samples were detected using a peptide nucleic acid (PNA) PCR mixture (20 µL) containing 7 µL of extracted DNA, 3 µL of PNA mixture, and 10 µL of KRAS PNA premixture, which were provided in the PNAClamp™ KRAS Mutation Detection Kit (Panagene, Daejeon, South Korea). Real-time PCR was performed using the StepOnePlus Real-Time PCR System (Applied Biosystems). The PCR conditions were as follows: 94 °C for 5 min, followed by 40 cycles of 94 °C for 30 s, 70 °C for 20 s, 63 °C for 30 s, and 72 °C for 30 s.

### Statistical analyses

All statistical analyses were performed using R software (version 4.1.0; http://cran.r-project.org/). The Wilcoxon rank-sum tests and Kruskal-Willis tests were used to determine correlations between miRNA expression levels and the clinicopathological features of the patients with CRC. The maximal Χ^2^ method was used to define the optimal cut-off values for continuous variables. Kaplan–Meier survival analysis was performed to determine the associations of variables with survival. Spearman’s correlation coefficient was used to determine the relationship between miRNAs and the tumor immune microenvironment in the tumor tissues. To compare immune cell densities or miRNA expression levels between the two groups, Wilcoxon rank-sum tests were performed after verifying that the groups in the test did not follow normal distributions. The tests were unpaired, except for the comparison of miRNA expression levels between tumor and normal tissues, which was performed using a paired test. P values < 0.05 were regarded as statistically significant.

## Results

### Evaluation of miRNA expression in tissue samples using ddPCR

**Figure 1:**
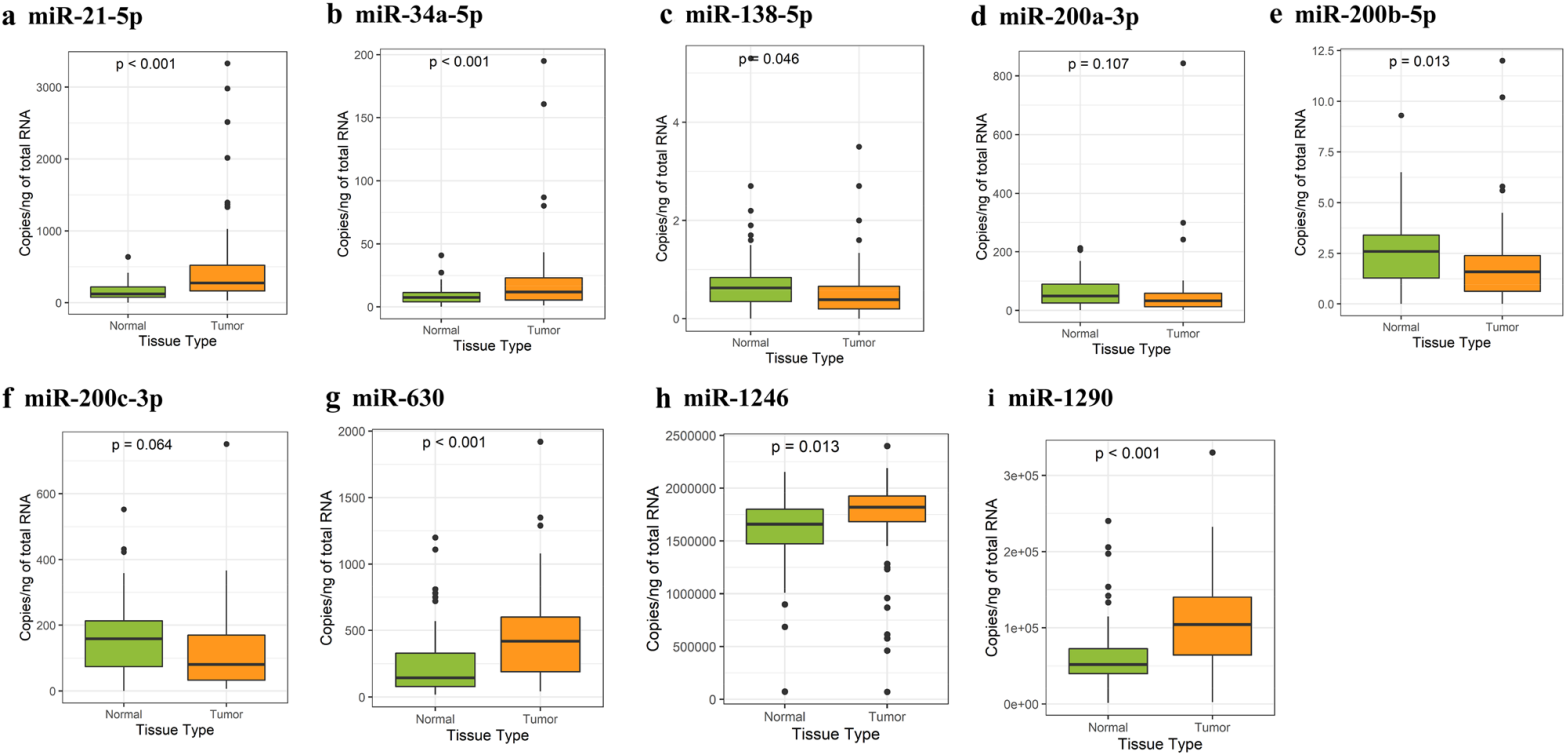
miRNA expression levels in tumor and normal tissues from CRC patients. Tumor tissues had high expression of miR-21-5p, 34a-5p, 630, 1246, and 1290 compared to normal tissues (**a**, **b**, **g**–**i**). The expression levels of miR-138-5p, 200a-3p, 200b-5p, and 200c-3p were lower in tumor tissues than in normal tissues (**c**–**f**).

We investigated nine different miRNAs that were previously reported to be related to EMT in solid tumors, namely miR-21-5p, 34a-5p, 138-5p, 200a-3p, 200b-5p, 200c-3p, 630, 1246, and 1290. The miRNA expression levels in paired normal and cancer tissues were measured using ddPCR. Compared to expression levels in matched normal tissues, expression levels of most miRNAs in tumor tissues showed statistically significant differences (Fig. 1). miR-21-5p, 34a-5p, 630, 1246, and 1290 showed significantly higher expression in tumor tissues than in normal tissue (*P* < 0.05). miR-138-5p and miR-200b-5p showed lower expression in tumor tissues than in normal tissues (*P* < 0.05), but the number of miR-138-5p and miR-200b-5p copies was small (mean, < 5 copies/ng).

### Relationships between tissue miRNAs and the tumor immune microenvironment

**Figure 2:**
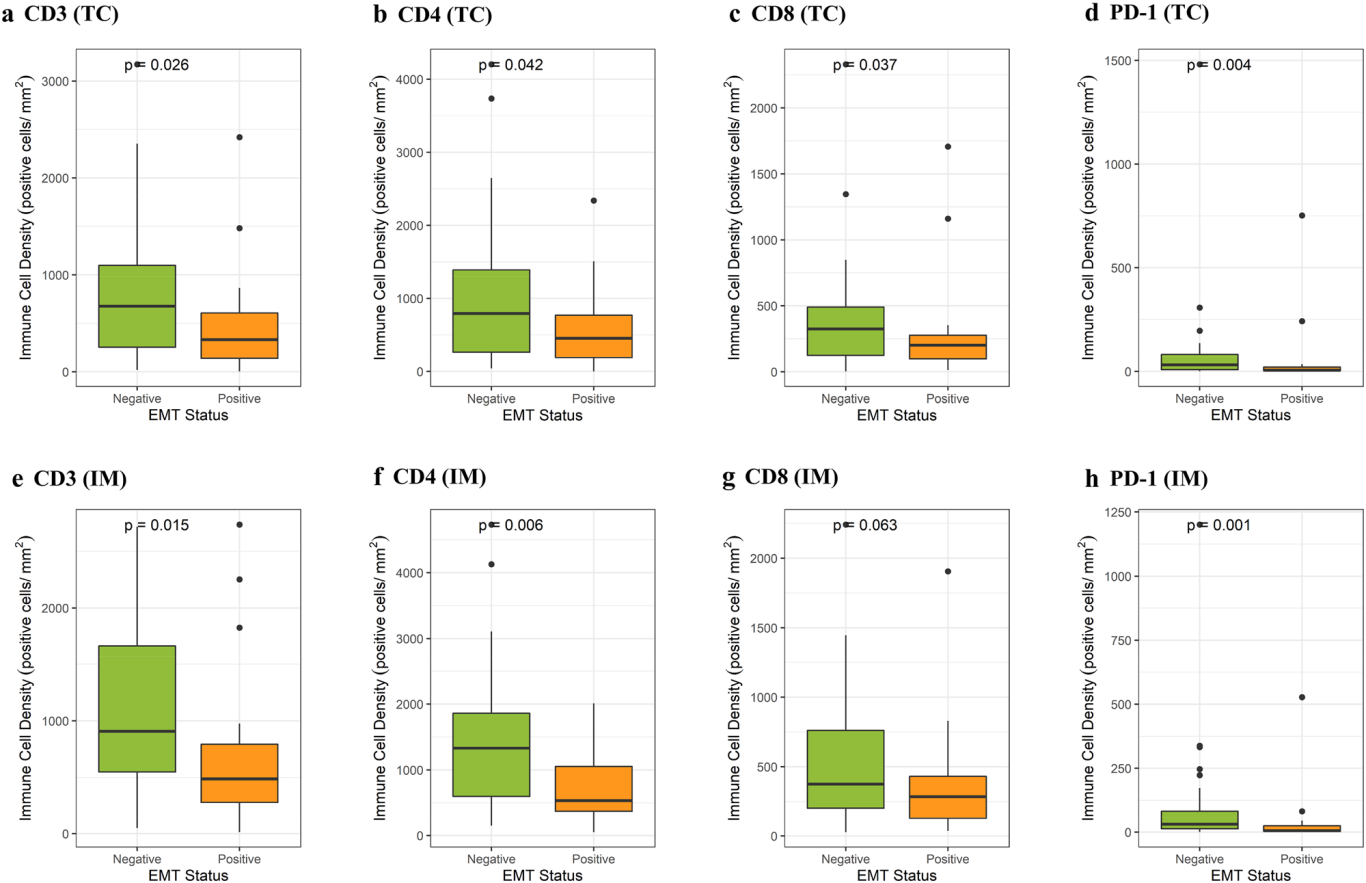
EMT status was associated with a low density of tumor-infiltrating immune cells (CD3, CD4, CD8) and the immune checkpoint protein PD-1 in TC (**a**–**d**) and IM (**e**–**h**).

After classifying the patients into two groups on the basis of their EMT status, we observed a significant difference between the groups in terms of their T cell densities in both TC and IM tissues. T cell markers (CD3, CD4, and CD8) were expressed at a lower density in CRCs with the EMT phenotype in both TC and IM tissues (Fig. 2a–f,h, *P* < 0.05; Fig. 2g, *P* = 0.063). Similarly, the EMT phenotype was associated with lower density of PD-1 in both TC (Fig. 2d; *P* = 0.031) and IM (Fig. 2h; *P* = 0.026) tissues. However, no significant correlation was found between the EMT status and a PD-L1 CPS of ≥ 1 (data not shown; *P* = 0.294 and 0.923, respectively).

**Figure 3:**
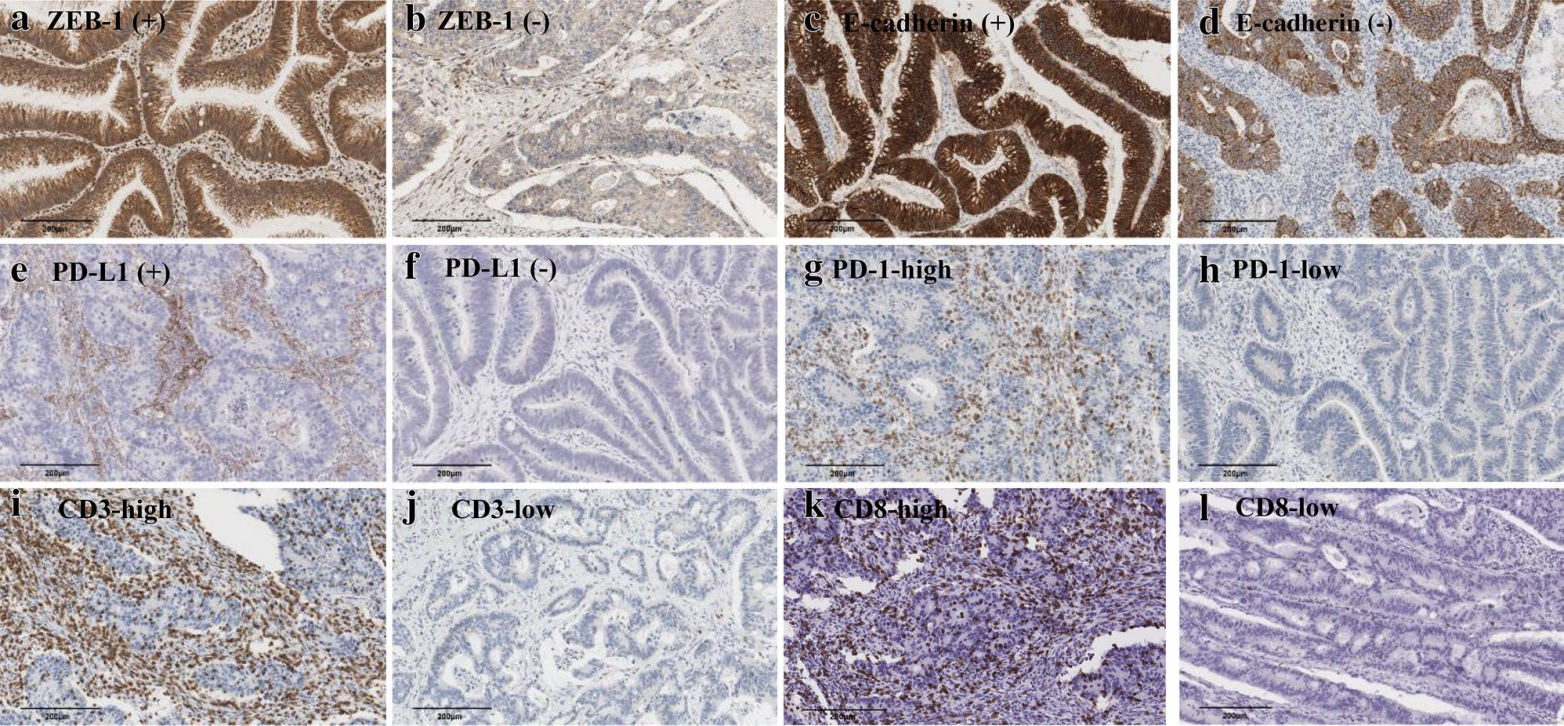
Representative images of immunohistochemistry of EMT markers (ZEB-1 and E-cadherin), immune checkpoints (PD-L1 and PD-1), and immune cell markers (CD3 and CD8) in tumor tissues.

The tissue expression levels of the nine miRNAs included in this study did not show statistically significant correlations with the EMT status of the patient (*P* > 0.05; data not shown). We investigated correlations between miRNA expression levels and tumor-infiltrating immune cell densities in tumor tissues (Fig. 3). The expression levels of miR-21-5p, miR-34a-5p, miR-200a-3p, and miR-200c-3p were negatively correlated with the densities of most tumor-infiltrating immune cells in both TC and IM tissues, as shown in Table 2. The results suggest weak but statistically significant (*P* < 0.05) correlations^28^; exact correlation coefficients are recorded in Table 2. Specifically, the CD8-positive T cell density was inversely correlated with miR-21-5p, miR-34a-5p, miR-200a-3p, and miR-200c-3p levels in IM tissues.

**Table 2:** Correlation coefficients between microRNA expression and tumor immune microenvironment (immune cell and immune checkpoint) in tumor center and invasive margin.

		Immune cell					Immune checkpoint	
		CD3	CD4	CD8	CD68	Foxp3	PD-1	PD-L1
Tumor center	miR-21-5p	− 0.247*	− 0.261*	− 0.238	− 0.127	− 0.145	− 0.256*	− 0.038
	miR-34a-5p	− 0.165	− 0.187	− 0.116	− 0.197	− 0.051	− 0.168	− 0.049
	miR-200a-3p	− 0.143	− 0.156	− 0.208	− 0.24	− 0.087	− 0.069	− 0.143
	miR-200c-3p	− 0.262*	− 0.252*	− 0.293*	− 0.281*	− 0.222	− 0.145	− 0.195
	miR-630	− 0.144	− 0.087	− 0.157	0.264*	0.093	− 0.221	0.056
	miR-1246	− 0.01	0.138	0.007	0.216	− 0.059	0.008	0.152
	miR-1290	− 0.083	0.012	0.033	0.226	0.012	0.069	0.082
Invasive margin	miR-21-5p	− 0.336**	− 0.335**	− 0.351**	− 0.259*	− 0.236	− 0.371**	− 0.21
	miR-34a-5p	− 0.279*	− 0.329**	− 0.273*	− 0.263*	− 0.26*	− 0.261*	− 0.179
	miR-200a-3p	− 0.246*	− 0.277*	− 0.265*	− 0.283*	− 0.246*	− 0.114	− 0.257*
	miR-200c-3p	− 0.293*	− 0.256*	− 0.327**	− 0.251*	− 0.287*	− 0.187	− 0.338**
	miR-630	− 0.153	− 0.1	− 0.193	0.051	0.123	− 0.16	− 0.09
	miR-1246	0.1	0.144	− 0.018	0.047	− 0.015	0.118	0.012
	miR-1290	0.075	0.003	0.07	0.135	0.059	0.212	0.031

Data are presented as Spearman’s correlation coefficient.

*P-value < 0.05, **P-value < 0.01.

Correlations with immune checkpoint markers were also investigated. The PD-1 positive immune cell density was negatively correlated with miR-21-5p (*P* = 0.002) and miR-34a-5p (*P* = 0.035) expression in IM tissues. miR-200c-3p expression showed a negative relationship with a PD-L1 CPS of ≥ 1 in IM tissues (Wilcoxon test, *P* < 0.05).

### Evaluation of miR-630, 1246, and 1290 expression in plasma samples

Because the quantity of nucleic acids in cell-free plasma is generally very low, we chose three miRNAs, which had tissue expression levels that suggested relatively abundant quantities, for further evaluation in plasma samples (Fig. 1). Specifically, we measured the expression levels of miR-630, 1246, and 1290 in cell-free plasma from healthy individuals and patients with CRC. The plasma samples were analyzed in triplicate by ddPCR to validate the reproducibility of the results. The intraclass correlations (ICCs) for all samples were > 0.950 except for miR-630 expression in healthy individuals (ICC = 0.514), suggesting that the results were highly reproducible (Additional file 2).

The plasma expression levels of miR-1246 and 1290 in patients with stage II–IV CRC were significantly higher than those in healthy volunteers (*P* < 0.05). The miR-630, miR-1246, and miR-1290 expression levels in patients with stage I CRC did not differ significantly from those in healthy volunteers (Additional file 3). Besides, the plasma levels of miR-640, 1246, and 1290 did not show statistically significant correlations with tumor-infiltrating immune cell densities or immune checkpoint markers (data not shown).

### Clinicopathologic correlations among EMT, tumor immune responses, and miRNAs

As expected, patients with CRC and the EMT phenotype had significantly worse OS (Additional file 4a; *P* < 0.001). PD-L1 positivity in IM and TC tissues predicted significantly better OS (Additional file 4b and 4c; *P* = 0.012 and 0.024, respectively). In addition, the high density of PD-1-positive immune cells in both IM and TC tissues was strongly associated with better OS (Additional file 4d and 4e; *P* < 0.001 and 0.002, respectively).

**Figure 4:**
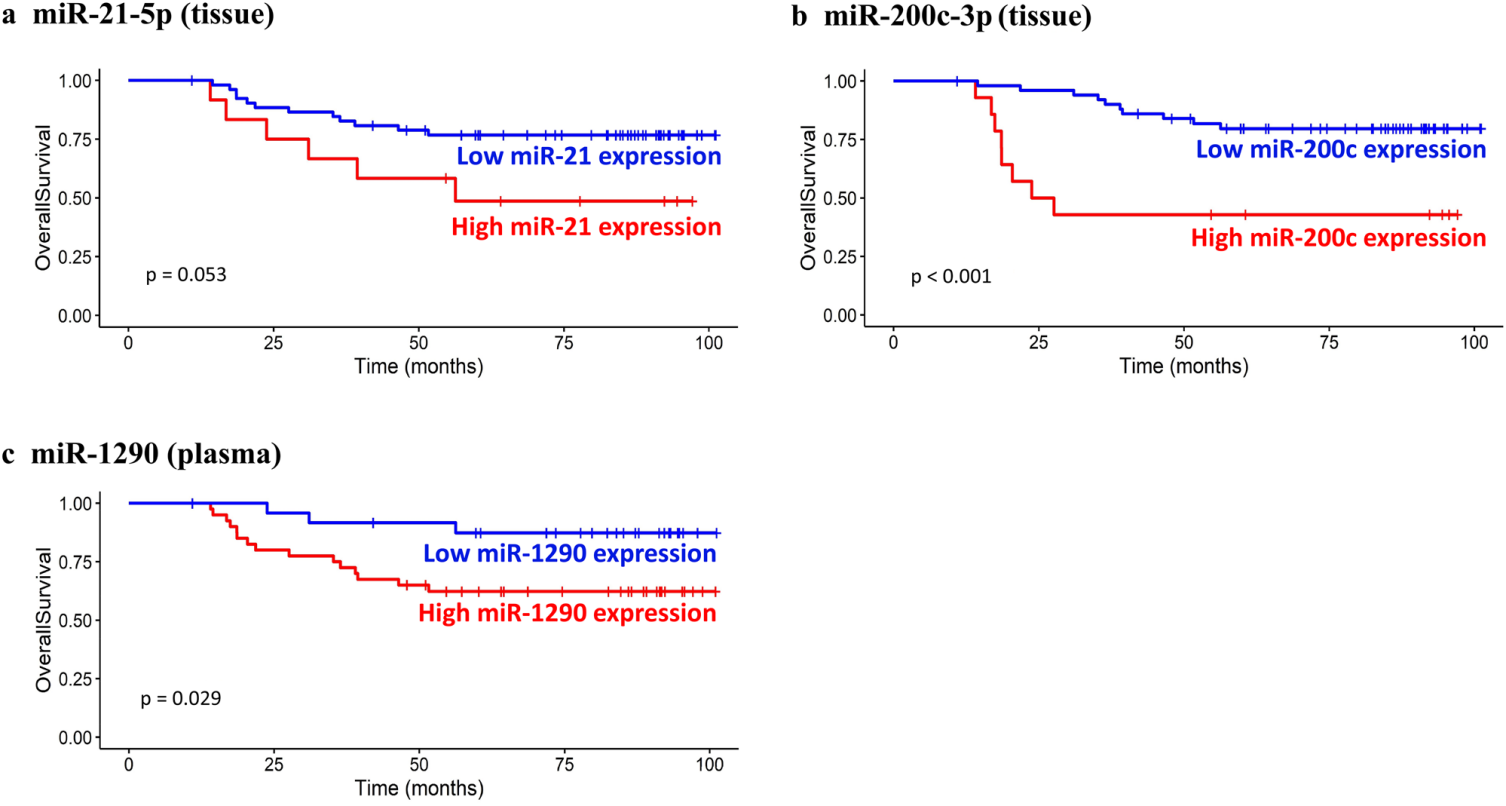
Kaplan–Meier survival analysis of the relationships between miRNA expression levels and OS. The expression level of miR-21-5p was (**a**) correlated with the patient OS but with borderline statistical significance. Patients with high miR-200c-3p expression in tissues (**b**) and high miR-1290 expression in plasma (**c**) had lower OS rates than patients with low expression of these miRNAs.

Table 3 summarizes the correlations between clinicopathologic parameters and miRNA expression levels. Higher expression levels of miR-21-5p and 200c-3p were observed in CRC cases with lymphatic invasion and an advanced tumor–node–metastasis (TNM) stage (*P* < 0.05) and were associated with worse OS (Fig. 4a and b; *P* = 0.053 and < 0.001, respectively). No significant difference in miRNA expression levels was found, according to the *KRAS* mutational status (*P* > 0.05).

**Table 3:** Relationship between the concentration of tissue microRNA-21-5p, 200c-3p, and plasma microRNA-1290 and clinicopathologic features.

Variable	T-miR-21-5p		T-miR-200c-3p		P-miR-1290	
	Median (range)	*P* value	Median (range)	*P* value	Median (range)	*P* value
	(10^4^ × copies/ng)		(10^4^ × copies/ng)		(copies/µL)	
**Sex**		0.198		0.182		0.548
Male	6.42 (0.909–99.9)		2.205 (0.222–22.53)		2.25 (0.617–8.1)	
Female	8.76 (0.903–41.82)		3.45 (0.195–10.47)		1.73 (0.837–7.067)	
**Tumor size**		0.795		0.358		0.369
< 5 cm	8.385 (0.903–75.39)		2.124 (0.195–10.47)		1.85 (0.653–8.1)	
≥ 5 cm	7.2 (1.413–99.9)		2.496 (0.222–22.53)		2.43 (0.617–6.533)	
**Histologic grade**		0.170		0.774		0.117
WD	4.11 (0.909–12.48)		2.711 (0.393–8.91)		1.258 (0.837–6.433)	
MD	8.37 (0.903–99.9)		2.43 (0.195–22.53)		2.067 (0.617–8.1)	
PD	11.28 (0.975–41.82)		2.385 (0.339–10.47)		3.233 (1.733–5.933)	
**Lymphatic invasion**		0.002**		< 0.001**		0.884
Absent	6.06 (0.903–75.39)		1.809 (0.195–8.91)		2.067 (0.617–8.1)	
Present	13.305 (3.06–99.9)		5.34 (0.234–22.53)		2.067 (0.75–5.933)	
**Venous invasion**		0.204		0.094		0.469
Absent	7.98 (0.903–89.4)		2.28 (0.195–11.01)		1.933 (0.617–8.1)	
Present	10.23 (2.97–99.9)		5.235 (0.228–22.53)		2.33(0.75–6.533)	
**Perineural invasion**		0.068		0.941		0.426
Absent	7.05 (0.903–60.48)		2.295 (0.195–9.6)		2.1 (0.617–7.067)	
Present	10.23 (1.107–99.9)		2.493 (0.222–22.53)		1.833(0.75–8.1)	
**TNM staging**		0.010**		0.007**		< 0.001**
I	6.27 (0.903–60.48)		1.398 (0.36–8.91)		1.23 (0.653–4.067)	
II	5.595 (1.107–28.02)		1.737 (0.195–6.24)		3.7 (1.223–8.1)	
III	10.92 (0.975–41.82)		2.285 (0.228–9.6)		1.4 (0.617–6.533)	
IV	12.21 (5.52–99.9)		5.79 (0.24–22.53)		2.287 (0.75–5.733)	
**Location**		0.577		0.022*		0.667
Right	6.885 (0.975–41.82)		5.55 (0.339–10.47)		2.117 (0.837–5.933)	
Left/rectum	8.4 (0.903–99.9)		2.13 (0.195–22.53)		1.933 (0.617–8.1)	
**KRAS**		0.665		0.943		0.431
Wild	8.175 (0.909–89.4)		2.204 (0.225–11.01)		1.683 (0.653–8.1)	
Mutant	8.28 (0.903–99.9)		2.76 (0.195–22.53)		2.1 (0.617–7.067)	

Data are presented as Median (range).

*P-value < 0.05, **P-value < 0.01.

T-, tissue; *P*- plasma.

*SD* standard deviation, *WD* well differentiated, *MD* moderately differentiated, *PD* poorly differentiated.

High plasma expression of miR-1290 showed a strong association with an advanced TNM stage (Table 3; *P* < 0.001). Furthermore, high plasma expression of miR-1290 was associated with worse OS (Fig. 4c; *P* = 0.029).

## Discussion

Calin et al. suggested the role of miRNAs in cancer in 2002^29^. miRNAs were established as cancer biomarkers in 2008 when Lawrie et al. identified miRNAs with diagnostic and prognostic value in diffuse large B-cell lymphoma^30^. miRNAs have many advantages as biomarkers, including easy extraction through liquid biopsies and high tissue-type specificity. Many researchers have attempted to establish new miRNAs as biomarkers for various diseases, with promising results, although such research is still in the early stages. For example, data from a previous study suggested that circulating miR-200c-3p may be useful as a diagnostic and prognostic biomarker for gastric cancer^31^. The results of another study suggested miR-148a as a biomarker for predicting the efficacy of chemotherapy in patients with advanced colorectal cancer^32^. Using such biomarkers may enable the customization of treatment strategies for individuals, which holds implications in personalized medicine.

Similar to some previous studies measuring multiple miRNA expression levels^33^, we also observed a great variance in the detected amount of miRNA molecules in this study. For example, tissue levels of miR-138-5p and miR-200b-5p were detected at amounts of fewer than 5 copies/ng of RNA in both normal and tumor tissues; therefore, the difference between paired tissues was small in scale although statistically significant (Fig. 1). However, tissue levels of miR-1246 and miR-1290 were detected at amounts of more than 1 × 10^6^ and 1 × 10^4^ copies/ng of RNA, respectively; consequently, the observed difference between paired tissues was greater in scale while statistical significance was comparative (Fig. 1). As the biological relevance of miRNAs as potential biomarkers includes not only the statistical significance but also the actual detected amount, the quantitative analysis provided by this study aids in the assessment of the potential clinical utility of the investigated miRNAs.

Our results suggest that tissue levels of miR-200c-3p and circulating levels of miR-1290 are potential prognostic biomarkers for CRC. In addition, the high expression level of each miRNA was associated with worse OS. Furthermore, as suggested by our current results and previous findings, such associations may reflect the key roles played by miR-200c-3p and miR-1290 in the regulation of EMT-immune crosstalk in CRC.

One seemingly counterintuitive result is that while the expression level of miR-200c-3p is lower in tumor tissue, patients with a high expression level of miR-200c-3p show poor survival. Previous studies suggest that the switch between EMT and mesenchymal-epithelial transition (MET) is a transient and dynamic process wherein EMT plays a critical role in the first stages of metastasis, such as tumor cell dissemination, while MET drives the later stages of metastasis, such as the colonization of the metastatic site^34^. miR-200c-3p downregulation is associated with EMT and miR-200c-3p overexpression is associated with MET, which may partially explain the seemingly unreasonable observation: the EMT-related expression pattern reflects the metastatic nature of the tumor tissue, but the MET-related expression pattern is associated with poorer survival owing to its role in more advanced metastatic stages. However, such an explanation is limited. Our current understanding of the role of miRNAs in cancer progression is rather oversimplified, where the complex mechanisms of multiple miRNAs regulating the expression of various genes are not thoroughly reflected. In fact, previous studies on the biomarker potential of miR-200c-3p in various cancer types have produced controversial results^35,36^. A more sophisticated understanding of the workings of miRNAs in the regulation of cancer progression is necessary for a more exhaustive explanation.

Crosstalk between EMT and the tumor immune microenvironment has widely been suggested for various types of tumors. In an ovarian carcinoma study, the mesenchymal subtype with an EMT-related gene signature correlated with the lower density of CD8-positive tumor-infiltrating lymphocytes^37^. Altered expression of EMT markers was associated with decreased tumor infiltration of CD4- and CD8-positive T cells in a study of non-small cell lung cancer^38^ and with upregulated inhibitory immune checkpoint molecules such as PD-L1 in a study of lung adenocarcinoma^39,40^. Such results indicate that EMT is a process involving evasion of the host immune system, and our results demonstrate a similar trend with CRC. The EMT statuses of the patients were highly correlated with a low density of tumor-infiltrating CD3-, CD4-, and CD8-positive lymphocytes. Similarly, low densities of PD-1-positive cells in both TC and IM tissues were correlated with an EMT phenotype in CRC patients. These results not only clarify EMT as an immune-related process but also hold implications for immunotherapy, as data from many previous studies have identified the tumor immune microenvironment as a key factor in predicting and explaining the success of immunotherapy in many cancer types^41–43^. A deeper understanding of the relationship between EMT and the tumor immune microenvironment may lead to refined immunotherapies.

We also identified miRNA markers associated with the EMT phenotype that had effects on tumor immune microenvironment. Notably, tissue levels of miR-200c-3p were negatively correlated with cells positive for immune cell markers such as CD3, CD4, and CD8 and negatively correlated with cells positive for the immune checkpoint marker PD-L1. These results agree with data from previous functional studies of the miR-200 family in regulating the immune system^44^, which may explain the worse OS observed in patients with high miR-200c-3p expression. Similarly, the circulating plasma levels of miR-1290 showed a negative association with CD3-, CD8-, and PD-1-positive cells. Considering that recent findings elucidated the role of miR-1290 in the immune escape of cancer cells in gastric cancer^45^, this finding may explain the worse OS observed in patients with high plasma levels of miR-1290.

This study does have some limitations. Notably, this study was conducted with a retrospective cohort at a single institute, and only 65 samples from patients with CRC were analyzed. Therefore, the potential utility of miRNAs suggested by this study as biomarkers needs to be further validated in an independent cohort as well as in a multi-centered study of a larger scale.

In conclusion, we identified tissue levels of miR-200c-3p and plasma levels of miR-1290 as potential prognostic markers of CRC, which may reflect functional associations of the miRNAs with EMT or the tumor immune microenvironment. Clinically, these miRNA markers could be used to accurately evaluate the prognosis and metastatic potential of CRC in an individual patient and to adjust the therapeutic strategy accordingly.

## Supplementary Information

Supplementary Information.

## Data Availability

The datasets supporting the conclusions of this article are included within the article and its additional files.

## Abbreviations

CD3: Cluster of differentiation 3
CD4: Cluster of differentiation 4
CD68: Cluster of differentiation 68
CD8: Cluster of differentiation 8
CPS: Combined positive score
CRC: Colorectal cancer
ddPCR: Droplet digital polymerase chain reaction
EMT: Epithelial–mesenchymal transition
Foxp3: Forkhead box P3
ICC: Intraclass correlation
IHC: Immunohistochemistry
IM: Invasive margin
miRNA: MicroRNA
OS: Overall survival
PCR: Polymerase chain reaction
PD-1: Programmed cell death protein 1
PD-L1: Programmed cell death ligand-1
TC: Tumor center
TMA: Tissue microarray
TNM: Tumor–node–metastasis
ZEB-1: Zinc finger E-box-binding homeobox 1
