# Supplementary material for: Tissue miR-200c-3p and circulating miR-1290 as potential prognostic biomarkers for colorectal cancer
Source: Sci Rep. 2022 Feb 10;12:2295. doi: 10.1038/s41598-022-06192-w (PMC8831555; doi:10.1038/s41598-022-06192-w)
Supplement: Supplementary file 1 — Supplementary Information. [file 41598_2022_6192_MOESM1_ESM.pptx]

## Slide 1
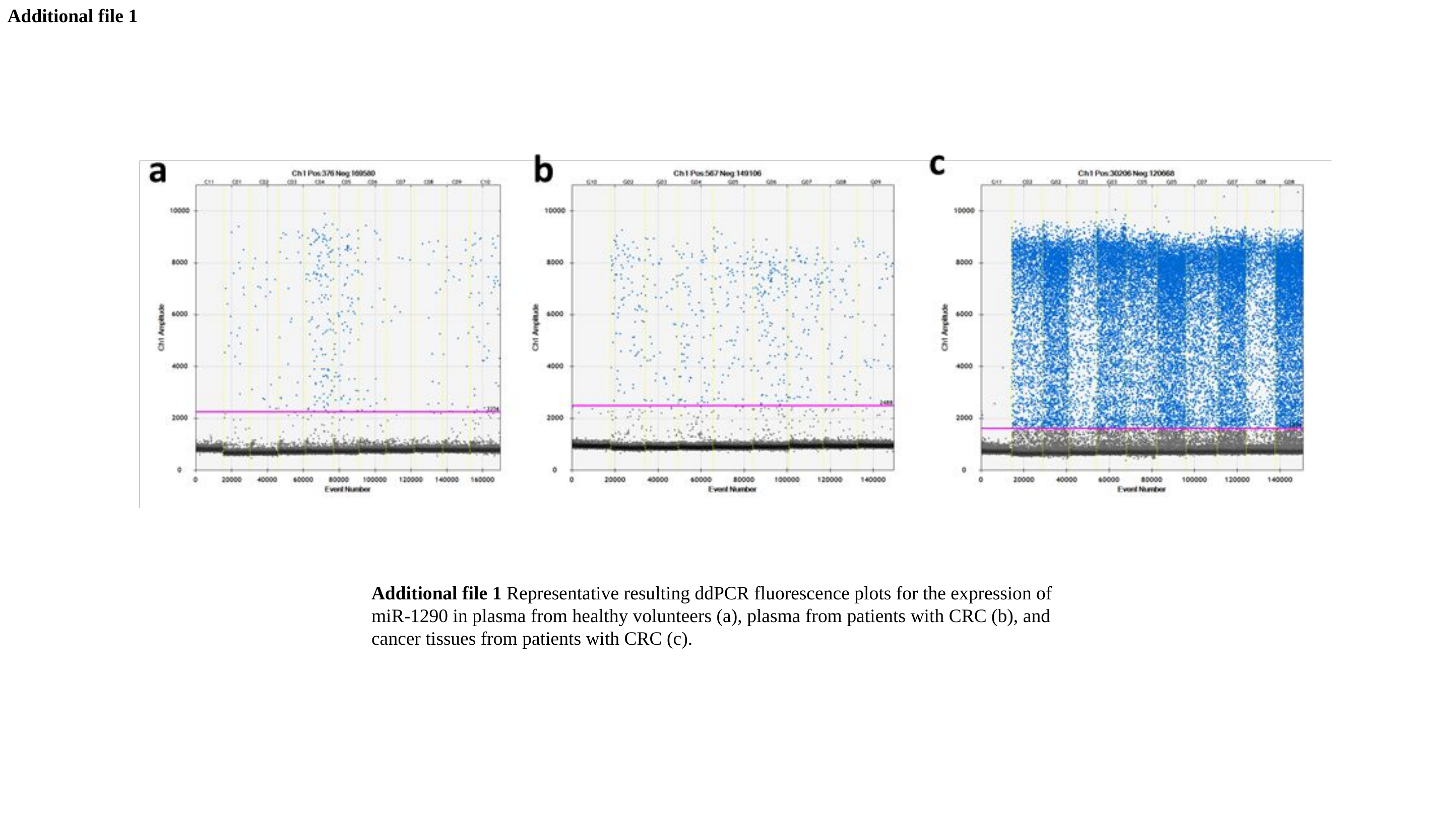

Additional file 1
Additional file 1 Representative resulting ddPCR fluorescence plots for the expression of miR-1290 in plasma from healthy volunteers (a), plasma from patients with CRC (b), and cancer tissues from patients with CRC (c).

## Slide 2
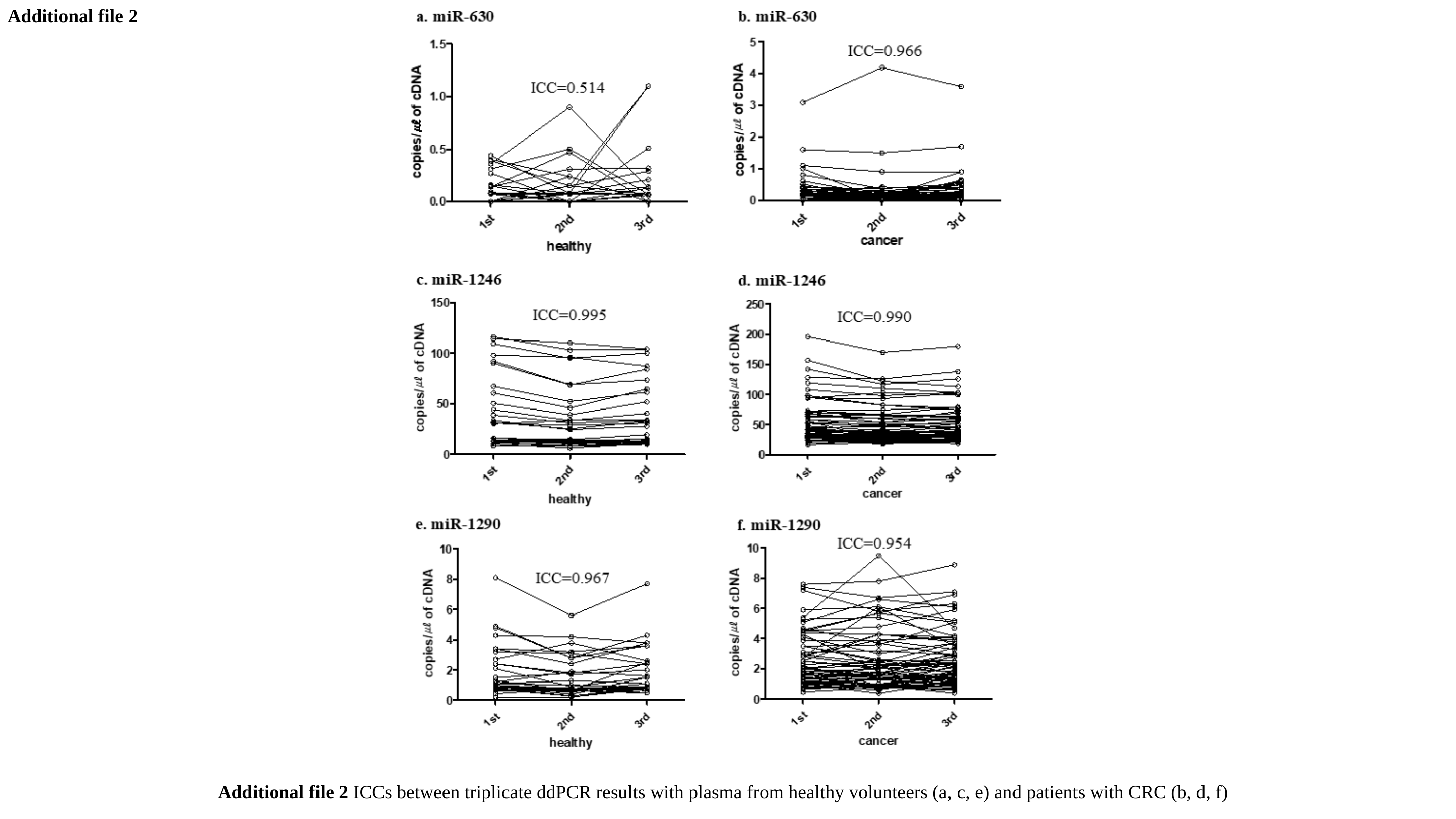

Additional file 2
Additional file 2 ICCs between triplicate ddPCR results with plasma from healthy volunteers (a, c, e) and patients with CRC (b, d, f)

## Slide 3
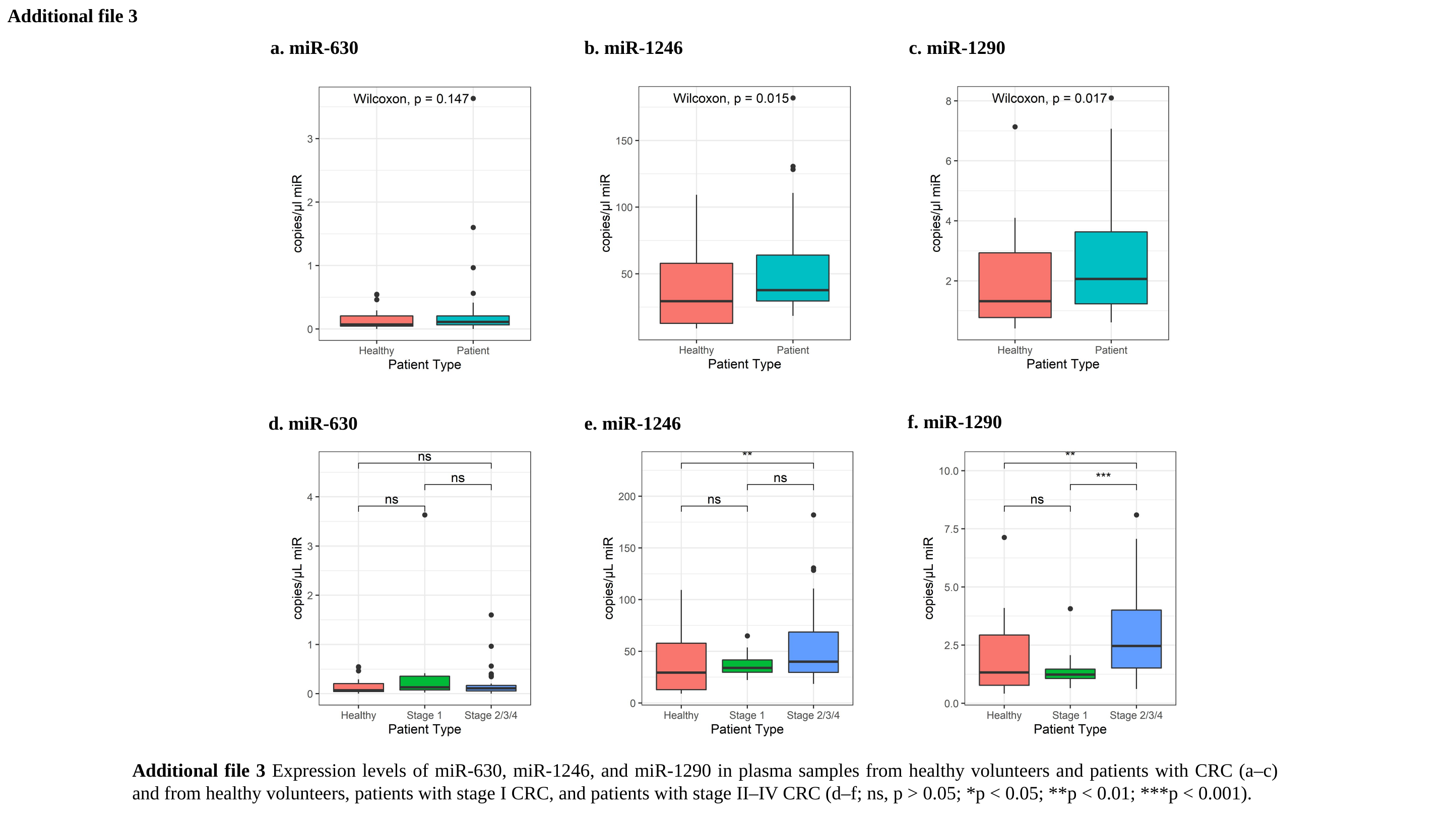

Additional file 3
a. miR-630
b. miR-1246
c. miR-1290
f. miR-1290
d. miR-630
e. miR-1246
Additional file 3 Expression levels of miR-630, miR-1246, and miR-1290 in plasma samples from healthy volunteers and patients with CRC (a–c) and from healthy volunteers, patients with stage I CRC, and patients with stage II–IV CRC (d–f; ns, p > 0.05; *p < 0.05; **p < 0.01; ***p < 0.001).

## Slide 4
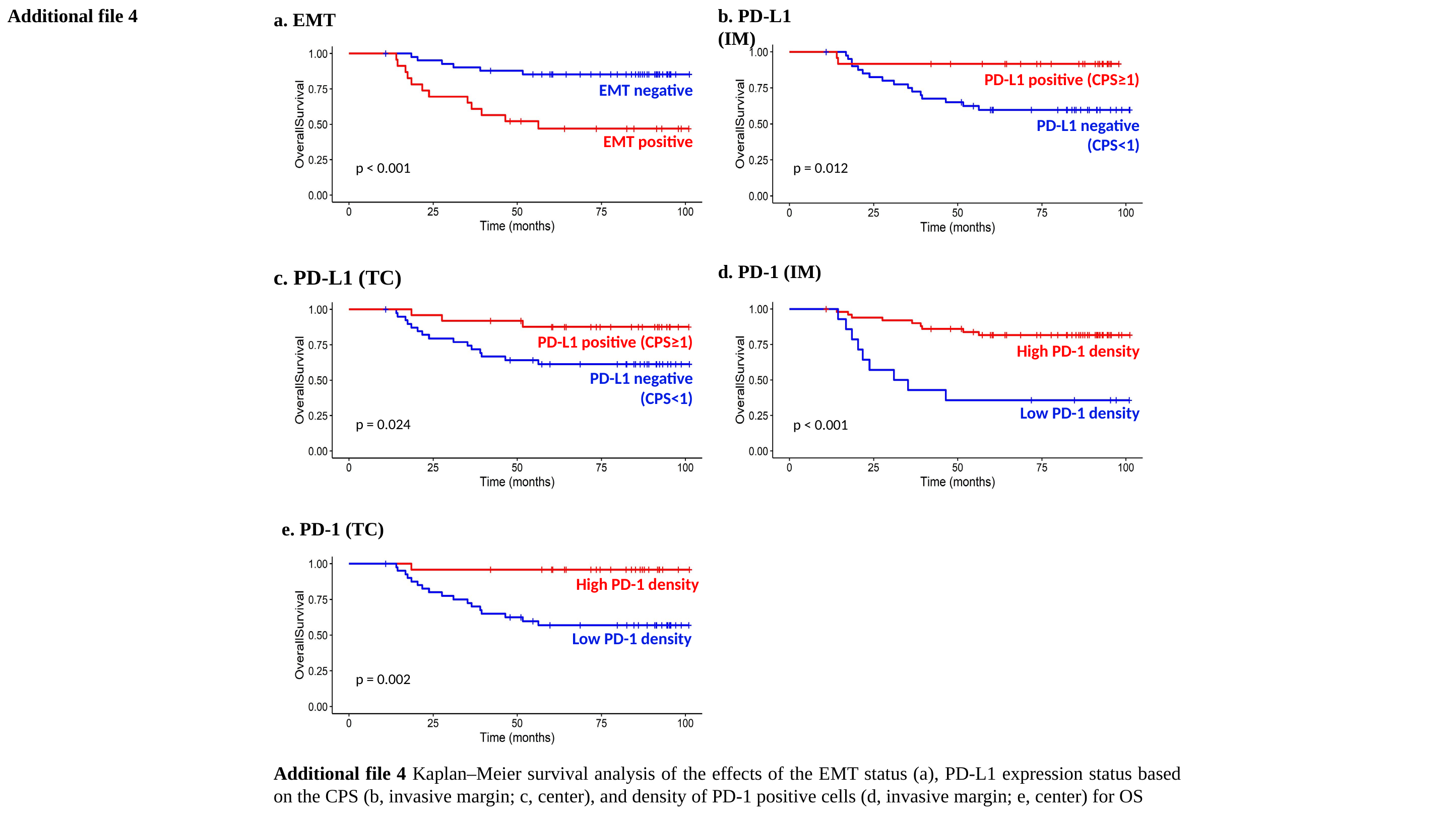

Additional file 4
b. PD-L1 (IM)
a. EMT
PD-L1 positive (CPS≥1)
PD-L1 negative (CPS<1)
EMT negative
EMT positive
d. PD-1 (IM)
c. PD-L1 (TC)
PD-L1 positive (CPS≥1)
PD-L1 negative (CPS<1)
High PD-1 density
Low PD-1 density
e. PD-1 (TC)
High PD-1 density
Low PD-1 density
p < 0.001
p = 0.012
p = 0.024
p < 0.001
p = 0.002
Additional file 4 Kaplan–Meier survival analysis of the effects of the EMT status (a), PD-L1 expression status based on the CPS (b, invasive margin; c, center), and density of PD-1 positive cells (d, invasive margin; e, center) for OS
